# Editorial and Miscellaneous

**Published:** 1865-09

**Authors:** 


### EDITORIAL AND MISCELLANEOUS.

### THE RUSH MEDICAL COLLEGE DISPENSARY.

This charity—established for the relief of those w ose lot embraces the multiplied misfortunes which follow in the train of indigence and disease—was, of kindred institutions, among the earliest founded in the city, and has been the means of lightening the burdens which have bowed down large numbers of poor, but many of them worthy people. Nor have its benefits been confined exclusively to these unfortunate classes, for indirectly they have reached the more highly favored and wealthy. The large number of prisoners of war confined at Camp Douglas, the troops necessarily stationed here for guard duty, and passing through in their transit to and from the front, made the small-pox for a time epidemic in the city, the municipal government in the meantime making no adequate provision to prevent the spread of the disease. Especially during this period the poor flocked to the Dispensary for vaccination, and undoubtedly many in this way escaped the loathsome and fatal disease, and thus the wealthy were protected by the prevention of the development of new centres of infection. The Dispensary is open every Monday, Wednesday and Saturday, in the College building. Dr. C. H. Duk has very kindly taken the principal charge of it during the past year, and has been untiring in his devotion to the interests of those who have presented themselves for treatment, as well as to the instruction of students in attendance. It is visited by several thousand patients annually, which brings together a greater variety of rare and interesting cases than any physician is likely to see in private practice.

During the summer months its clinics are attended by students who are pursuing their studies in the city, while in the lecture season the most interesting cases are brought before the class. Prof. Brainard prescribes for and operates on the surgical cases, and among the operations thus witnessed by the class last winter were several of lithotomy, amputations, removal of tumors, operations for hare-lip, varicocele, reduction of dislocations, dressing of fractures, extirpation of superior and inferior maxillary bones, &c., &c.

### DEMONSTRATOR OF RUSH MEDICAL COLLEGE.

Dr. Lynn has resigned his place as Demonstrator in this institution, carrying with him the esteem and kind wishes of the Faculty, with whom he has been associated in a very responsible position during the last three years. The place has been filled by the appointment of Dr. Edwin Powell, well known to the Alumni of the school from having occupied the same station a number of years with marked ability. On the breaking out of the war, he gave up his position for service in the army, where he made a record as an operator, and as chief Surgeon of one of our most important and best conducted hospitals, of which his Alma Mater may well be proud. Dr. Powell's numerous friends, both in civil and military life, will certainly congratulate the school that the place made vacant has been so worthily filled.

MARRIED—At Bedford, Lawrence Co., Ind., July 30th, by the Rev. Wm. F. Harned, Jos. H. WILSON, M. D., and JENNIE A. HARRIS, both of Marion, Linn Co., Iowa.

